# Disulfiram, an aldehyde dehydrogenase inhibitor, works as a potent drug against sepsis and cancer via NETosis, pyroptosis, apoptosis, ferroptosis, and cuproptosis

**DOI:** 10.1097/BS9.0000000000000117

**Published:** 2022-07-01

**Authors:** Dingrui Nie, Cunte Chen, Yangqiu Li, Chengwu Zeng

**Affiliations:** a Key Laboratory for Regenerative Medicine of Ministry of Education, Institute of Hematology, School of Medicine, Jinan University, Guangzhou, China

**Keywords:** Apoptosis, Cuproptosis, Disulfiram, Ferroptosis, Sepsis, NETosis, Pyroptosis

## Abstract

Regulated cell death (RCD) is essential for maintaining cell homeostasis and preventing diseases. Besides classical apoptosis, several novel nonapoptotic forms of RCD including NETosis, pyroptosis, ferroptosis, and cuproptosis have been reported and are increasingly being implicated in various cancers and inflammation. Disulfiram (DSF), an aldehyde dehydrogenase inhibitor, has been used clinically for decades as an anti-alcoholic drug. New studies have shown that DSF possesses potent anti-inflammatory and anti-cancer effects by regulating these new types of RCD. Here, we summarize the mechanisms and discuss the potential application of DSF in the treatment of cancers and inflammatory diseases.

Disulfiram (DSF), an aldehyde dehydrogenase inhibitor that affects alcohol metabolism, was approved by the US Food and Drug Administration (FDA) in 1951 for the treatment of alcoholism.^1^ DSF has since been used clinically, and numerous studies and clinical trials have shown that it is a safe drug without significant side effects at the US FDA-recommended dosage.^2^ Importantly, recent studies have demonstrated that DSF possesses potent antiinflammatory and anticancer effects attributed to influencing several types of regulated cell death, such as NETosis, pyroptosis, apoptosis, ferroptosis, and cuproptosis (Fig. 1).

**Figure 1.**
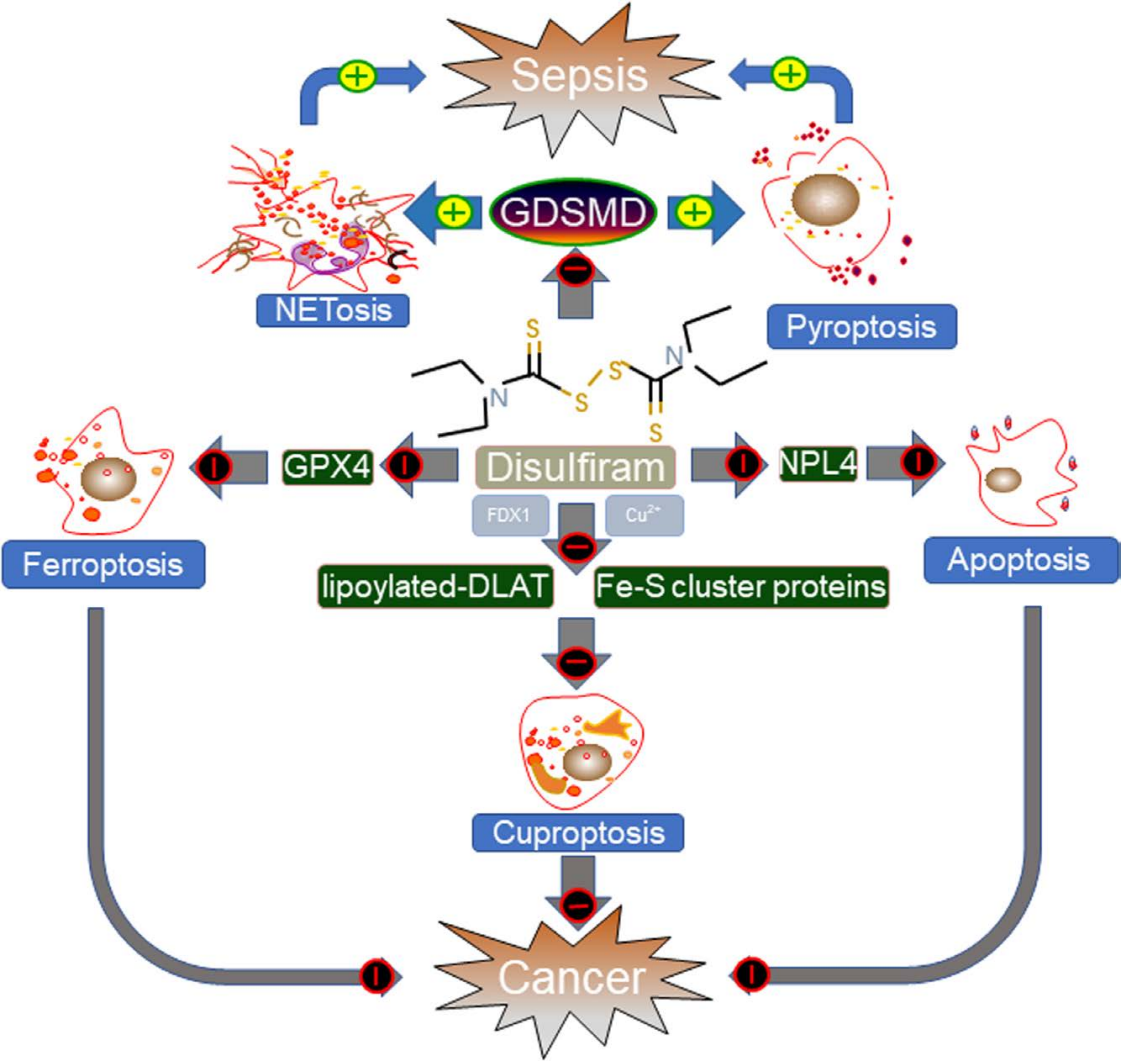
Antiinflammatory and anticancer mechanisms of DSF. DSF can significantly inhibit GSDMD to repress pyroptosis in macrophages and NETosis in neutrophils during sepsis. DSF triggers apoptosis via the p97 segregase adaptor NPL4. DSF can also induce ROS and downregulate the protein level of GPX4, leading to ferroptosis. The active form of DSF transports copper to intracellular compartments, and this intracellular copper accumulation induces cuproptosis through FDX1-mediated mitochondrial proteotoxic stress. DSF = disulfiram, FDX1 = ferredoxin 1, GSDMD = gasdermin D, GPX4 = glutathione peroxidase 4, ROS = reactive oxygen species.

Sepsis is a systemic host response to infection, which is a life-threatening condition and may lead to dysfunction in multiple organs. Dysregulation of the monocyte-macrophage and the granulocyte systems is responsible for sepsis progression.^3–5^ Previous reports have suggested that a gasdermin D (GSDMD)-dependent mechanism plays a central role in the physiopathology of sepsis. Recent studies have shown that DSF can significantly inhibit GSDMD to hinder the progression of pyroptosis in macrophages^6^ and the excessive formation of neutrophil extracellular traps (NETs) in neutrophils during sepsis.^7^ GSDMD is a pore-forming protein with a central executive role in inflammatory cell death called pyroptosis. In detail, inflammasomes activate caspase-1 to cleave GSDMD, and in turn, GSDMD is cleaved at the junction between the N-terminal domain (GSDMD-NT) and the autoinhibitory C-terminal domain (GSDMD-CT). GSDMD-NT binds to acidic phospholipids in the inner leaflet of the plasma membrane and oligomerizes to form pores that disrupt plasma membrane integrity, both enabling the release of the proinflammatory cytokines IL-1β and IL-18, which are processed by caspase-1, thereby inducing pyroptotic cell death.^8–10^ A previous study has demonstrated that DSF can inactivate reactive Cys residues by covalent modification.^11^ Wu et al further demonstrated both in vitro and In vivo that DSF curbs pyroptosis by modifying Cys191 in human GSDMD and Cys192 in mouse GSDMD to hinder the pore formation of NT-GSDMD rather than GSDMD cleavage or other earlier events.^6^ The same mechanism has also been verified in severe acute pancreatitis^12^ and gouty arthritis.^13^ The neutrophil cell death NETosis is a program for the formation of NETs to trap and kill microorganisms.^14^ NETosis is induced in response to infection and must be tightly regulated to prevent excessive tissue damage during sepsis. Recently, Silva et al^7^ also observed that the concentration of NETs positively correlates with the severity of sepsis. The authors demonstrated that DSF-mediated GSDMD inhibition in neutrophils efficiently abrogates NETosis, and treatment of septic mice with DSF inhibited systemic inflammation and vital-organ dysfunction and improved survival. In addition, a preclinical study found that DSF reduced NETs and protected against lung injury in a golden hamster model of COVID-19.^15^ Hence, GSDMD may be one of the key targets of DSF that limits both NETosis and pyroptosis. These studies on DSF in inflammation bring new clarity to the clinical treatment of sepsis and other inflammatory diseases.

It is important to note that DSF has anticancer activity in a range of solid and hematological malignancies.^16–18^ In contrast to its inhibition of NETosis and pyroptosis in sepsis, DSF has been shown to promote several other types of cell death in cancer cells, including apoptosis,^18^ ferroptosis,^19^ and cuproptosis.^20^ Apoptosis is a programmed cell death caused by the activation of exogenous signals such as Fas-FasL or the release of endogenous cytochrome C in cells to activate the caspase family. Inducing apoptosis has far-reaching significance in the treatment of malignancies.^21,22^ Skrott et al^18^ identified that CuET (bis-diethyldithiocarbamate-copper), a metabolite of DSF combined with copper, significantly inhibits human breast cancer in vitro and vivo by binding NPL4 (NPL4 homolog, ubiquitin recognition factor) and inducing its aggregation; this results in a heat-shock response and cell death. Consistent with their results, our recent data revealed that DSF induces apoptosis and inhibits cell proliferation in cell lines of malignant T-cells and in primary T-cell acute lymphoblastic leukemia (T-ALL) cells by targeting the NPL4-mediated ubiquitin-proteasome pathway.^23^ Of note, Skrott et al^18^ demonstrated that CuET induces apoptosis in sensitive cell lines, whereas in most cell lines, CuET induces apoptosis-independent cell death. The detailed molecular mechanism and significance of the apoptosis-independent cell death induced by DSF remain obscure.

Accumulating evidence indicates that ferroptosis, an iron-dependent programmed cell death caused by unbalanced lipid redox metabolism,^24^ is one of the anticancer mechanisms of DSF. DSF can raise the level of reactive oxygen species (ROS) and downregulate the protein level of glutathione peroxidase 4 (GPX4) to induce ferroptosis in glioblastoma, and this phenomenon can be rescued by the ferroptosis inhibitor ferrostatin-1.^25^ It was also reported that the DSF/Cu complex suppresses the cell viability, migration, invasion, and angiogenesis of hepatocellular carcinoma (HCC) cell lines. The authors confirmed that this effect is related to the excessive level of free iron and lipid peroxidation, which subsequently induces the occurrence of ferroptosis. DSF/Cu treatment also leads to activation of the transcription factor NRF2, which plays a key role in counteracting oxidative stress. Recent studies have shown that NRF2 not only regulates the expression of antioxidant genes but also directly regulates the expression of iron metabolism and lipid oxidation-related genes.^26, 27^ Furthermore, the authors suggest that targeting NRF2 strengthens HCC cells that are more vulnerable to DSF/Cu induced ferroptosis.^19^

A recent study identified that intracellular copper overload triggers a novel form of cell death, called cuproptosis, which is mechanistically different from apoptosis and other types of regulated cell death. The authors identified ferredoxin 1 (FDX1) as playing a key role in copper-induced death. FDX1 is a reductase that can reduce Cu^2+^ to the more toxic form, Cu^+^. They also found that FDX1 is involved in protein lipoylation—a highly conserved lysine posttranslational modification of mitochondrial enzymes that is essential for mitochondrial metabolism. Furthermore, copper binds directly to lipoylated mitochondrial enzymes (especially dihydrolipoamide S-acetyltransferase) and interferes with iron-sulfur (Fe-S) cluster proteins in an FDX1-dependent manner. This results in lipoylated protein aggregation and subsequent Fe-S cluster protein degradation, which leads to mitochondrial proteotoxic stress and ultimately cell death.^20^ Notably, the authors also discovered that DSF is a potent copper ionophore, and that induced cell death depended on copper accumulation. This study suggests that DSF could potentially be used to treat cancer with high levels of lipoylated proteins.

Although many studies have identified that DSF has potential therapeutic activity against various types of cancers, the precise mechanisms remain to be explored further. Of particular importance in recent studies is that DSF has been shown to trigger ferroptosis and cuproptosis in several cancer cells. Chemotherapy resistance is a major challenge to the successful treatment of cancer. Of note, it has been reported that the survival of drug-tolerant persistent cancer cells is highly susceptible to ferroptosis inducers or copper ionophores.^20, 28^ Thus, triggering these novel forms of cell death represents a potential therapeutic approach for therapy-resistant cancer cells. The identification of FDA-approved DSF as a ferroptosis inducer and copper ionophore in cancer creates high expectations for the potential of this drug in cancer therapy based on the induction of ferroptosis or cuproptosis.
